# Behavior, protein, and dendritic changes after model traumatic brain injury and treatment with nanocoffee particles

**DOI:** 10.1186/s12868-019-0525-5

**Published:** 2019-08-22

**Authors:** Whitney A. Ratliff, Jessica N. Saykally, Ronald F. Mervis, Xiaoyang Lin, Chuanhai Cao, Bruce A. Citron

**Affiliations:** 1 Bay Pines VA Healthcare System, Research and Development, 151, Bldg. 22 Rm. 123, 10000 Bay Pines Blvd, Bay Pines, FL 33744 USA; 2 Department of Molecular Medicine, USF College of Medicine, 12901 Bruce B. Downs Blvd, MDC 7, Tampa, FL 33612 USA; 3 NeuroStructural Analytics, Inc, Columbus, OH USA; 4 Center for Aging and Brain Repair, Department of Neurosurgery and Brain Repair, University of South Florida Morsani College of Medicine, 2 Tampa General Circle, Tampa, FL 33606 USA; 5 The USF-Health Byrd Alzheimer’s Institute, College of Pharmacy, University of South Florida, 4001 E. Fletcher Ave, Tampa, FL 33613 USA; 6 Department of Pharmaceutical Sciences, USF College of Pharmacy, 12901 Bruce B. Downs Blvd, Tampa, FL 33612 USA; 7 VA New Jersey Health Care System, Research & Development, Bldg. 16, Rm. 16-176, 385 Tremont Ave, Mailstop 15, East Orange, NJ 07018 USA; 8 Department of Pharmacology, Physiology & Neuroscience, Rutgers-New Jersey Medical School, 185 South Orange Ave., Newark, NJ 07101 USA

**Keywords:** Traumatic brain injury, Closed head injury, Coffee, Caffeine, Golgi stain

## Abstract

**Background:**

Traumatic brain injury (TBI) is a widespread public health problem and a signature injury of our military in modern conflicts. Despite the long-term effects of even mild brain injuries, an effective treatment remains elusive. Coffee and several of its compounds, including caffeine, have been identified as having neuroprotective effects in studies of neurodegenerative disease. Given the molecular similarities between TBI and neurodegenerative disease, we have devised a study to test a nanocoffee extract in the treatment of a mouse model of mild TBI.

**Results:**

After a single injury and two subsequent injections of nanocoffee, we identified treatment as being associated with improved behavioral outcomes, favorable molecular signaling changes, and dendritic changes suggestive of improved neuronal health.

**Conclusions:**

We have identified coffee extracts as a potential viable multifaceted treatment approach to target the secondary injury associated with TBI.

## Background

In the United States it is estimated that 1.7 million people experience a traumatic brain injury (TBI) each year [1]. Our military personnel and athletes are at a particular risk, which has garnered great media attention in recent years [2, 3]. The vast majority of these TBIs are mild, however, even mild TBI (mTBI) can result in pathological changes and long-term cognitive deficits. While many mTBI patients’ symptoms resolve in days or weeks, it is estimated that approximately 10% suffer long-term complications. There is currently no effective treatment for these deficits [4–7]. One of the most common long-term problems encountered by TBI patients is memory impairment [8, 9]. One major contributing factor in this is likely damage to the hippocampus, which has been shown to be susceptible to mechanical injury [10]. These deficits have been successfully recapitulated in animal models of closed head injury, which have demonstrated loss of neurons in the hippocampus and cognitive deficits similar to human patients [11]. Despite the many challenges faced by TBI patients, there are currently no effective treatments.

TBI is also associated with an increased risk of neurodegenerative disease such as Alzheimer’s Disease (AD). One hallmark of neurodegenerative disease is the accumulation of protein aggregates, the same pathology can be seen many years after even a single TBI in patients. In particular, we see widespread hyperphosphorylated tau and amyloid-beta (Aβ) pathologies, similar to those seen in AD patients [12, 13], as well as TAR DNA protein 43 (TDP-43), which has been implicated in the pathology of Amyotrophic Lateral Sclerosis (ALS) [14, 15]. Understanding protein accumulation following TBI is important when considering the development of therapeutics or the identification of natural products to combat post-TBI deficits.

Human studies have shown that coffee and the caffeine contained therein can have neuroprotective effects and even therapeutic potential against AD. Epidemiologic studies have shown improved cognitive outcomes in normal aging adults who consume coffee/caffeine [16]. Moreover, coffee consumption in mid-life has been associated with a 65% decrease in the risk of developing AD [17] and AD patients have been found to have consumed less coffee in the 20 years prior to diagnosis than age matched controls [18]. Also, habitual coffee consummation can delay the onset of AD [19]. These protective effects may be due in part to the impact that coffee/caffeine has on protein accumulation and, in particular, Aβ accumulation.

Studies in animal models of AD shed some light on the molecular mechanisms behind the cognitive benefits of caffeine. AD transgenic mice given caffeine in their drinking water have been shown to display reduced memory impairment in old age [20] and caffeine administration has even been shown to reduce memory deficits which are already present in older AD transgenic mice [21]. These effects are likely due to the ability of caffeine to suppress β and γ-secretase, resulting in a decrease in Aβ production and lower levels of brain Aβ [20, 21]. In early development, exposure to caffeine has been shown to alter GABAergic and hippocampal networks [22]. Further in vitro study has shown that caffeine also increases levels of protein kinase A in the brain, decreasing the stimulation of β-secretase by reducing the Raf-1/NFκB inflammatory pathway. In addition, caffeine suppresses stimulation of γ-secretase through a reduction in GSK-3α in neuronal cell cultures [21]. Caffeine has also been shown to prevent neuronal death as a result of Aβ accumulation in cultured cerebellar neurons [23]. This may be the result of caffeine’s known role in activating the adenosine A_2A_ receptor, which has been shown to impact neurodegeneration [24, 25]. In addition to caffeine, coffee also contains a number of potentially beneficial compounds, which have also been shown to work synergistically with caffeine to provide beneficial cognitive effects [19].

In addition to standard behavioral testing and protein analysis, we have utilized a silver staining technique, Golgi staining, to investigate individual neurons following injury and treatment. This technique allows for the analysis of specific neurons separate from their environment and neighboring neurons. As a result, we are able to visualize changes on the cellular level, which would not be picked up by other imaging methods. We have been able to evaluate dendritic complexity and branching, as well as soma size and dendritic spines. Several previous studies have shown changes in these measures following TBI in a variety of injury models [15, 26–28]. Additionally, one study showed that caffeine consumption also had an impact on length, branching, and spine density within the hippocampi of rats, which was associated with a reduction in age-associated cognitive decline [29].

Given the molecular similarities between TBI and AD, it stands to reason that the neuroprotective effects of caffeine and other coffee components observed in AD may also translate to that of TBI. However, there has been little previous research investigating coffee’s effect on the pathogenesis of TBI. In order to better understand the mechanisms involved, our study investigates the molecular, neurostructural, and behavioral implications of treatment with a nanocoffee particle on mice receiving a mild TBI from a closed head injury.

## Materials and methods

### Animals

The animal protocol was approved by the Bay Pines VA Institutional Animal Care and Use Committees (IACUC) and performed in accordance with all institutional, agency, and governmental Animal Welfare Regulations. Male CD-1 mice at 6 weeks of age and weighing between 31 and 34 g were obtained from Harlan Laboratories (Indianapolis, IN). They were housed in our Veterinary Medical Unit (VMU) at 3–4 per cage in a 22 °C ± 0.5 °C temperature-controlled environment with a 12 h light/dark cycle. Food and water were available ad libitum and health checks were performed daily with weights taken weekly at a minimum. Power analysis (to 90%) was used to determine sample sizes for experimental and control groups. Animals were randomly assigned to numbered cages by VMU staff without input from the investigators. Each numbered cage was assigned to a group prior to investigators handling the mice. Mice were then individually numbered, and experiments were performed on mice in numbered order.

### Closed head injury

**Fig. 1:**
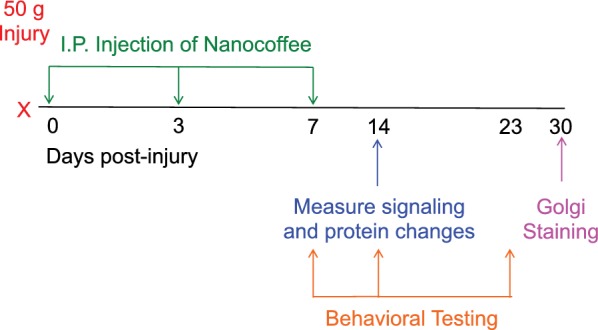
Closed head injury mouse model. The experimental timeline began with a 50 g weight dropped 80 cm onto the right side between the ear and eye followed by treatment with the nanocoffee compound initiated 30 min after injury and administered again 3- and 7-days post-injury. As shown, behavioral tests were performed 7, 14, and 23 days post-injury. Protein analysis was performed on brains collected 14 days post-injury. At 30 days post-injury, brains were collected and analyzed via Golgi stain

Traumatic brain injury (TBI) was induced using a concussive closed head injury, as described previously [11], which has been demonstrated to cause a mild injury (mTBI) [30]. Injuries were performed in the surgical suite of the Veterinary Medical Unit during mid-morning. Briefly, mice were anesthetized (anesthetic depth confirmed by toe pinch) with isoflurane and placed on a sponge, which allows for rotation of the head to inflict a diffuse concussive injury [31]. The injury apparatus was positioned directly over the head with the inner diameter of the tube (13 mm) spanning the right hemisphere caudal to the eye and rostral to the ear. A 50 g cylindrical weight was dropped 80 cm through the length of the tube in injured mice (*n *= 16). Sham mice (*n *= 16) did not receive weight drop portion of procedure, but were exposed to anesthesia and placed on the sponge for an equivalent period of time. Mice were allowed to recover from anesthesia in a heated chamber before being returned to the home cage. Injuries, treatments, and analyses are detailed in Fig. 1.

### Preparation and treatment with nanocoffee particles

40 g of dark roast coffee (Maxwell House, Kraft Foods, Chicago, IL) was added into 300 ml double-distilled water and brewed by heating to boiling and keeping at a boil for 2 min [19]. The brewed coffee was then filtered through a coffee filter to remove particles and coffee oil, and then the filtered coffee was sonicated at the highest power for 10 min. The sonicated coffee was checked for Nanocoffee particle formation and then aliquoted into a 25 ml per tube and stored at − 80 °C for future application. Following injury, mice receiving treatment were given an intraperitoneal injection containing 200 μl of the nanocoffee solution (100% saline vehicle was used as a vehicle). Injections were given at 30 min, 3 days, and 7 days post-injury to each mouse.

### Behavioral tests

An elevated plus maze test was done on a subset of animals (n = 7 for TBI Nanocoffee and Sham Vehicle groups and n = 6 for TBI Vehicle and Sham Nanocoffee groups) 7 days post-injury. The test involved placing the mouse in the center of an elevated platform with two open arms and two closed arms. The mouse was allowed to explore the apparatus for 5 min. Additionally, a novel object recognition test was done 14 and 23 days post-injury (n = 7 for all groups). On day one, the mouse was placed in the center of an open field and allowed to explore for 5 min. The following day, the mouse was placed in the same field containing two identical objects and allowed to explore for 5 min. The last day, one of the objects was replaced with a novel object and the mouse was given 5 min to explore. In both tests, the mouse’s movements were recorded and tracked using Stoelting Any-Maze video tracking software. All behavioral testing was performed in a behavioral testing suite in the Veterinary Medical Unit during mid-day.

### Protein analysis

Mice were humanely euthanized by cervical dislocation, followed by decapitation, and brain tissue was rapidly dissected while chilled on ice then snap frozen in liquid nitrogen (n = 7 for all groups). No anesthesia was used prior to cervical dislocation to avoid changes in regulatory factors that we are studying brought about by anesthesia. This exception was approved by the Bay Pines IACUC and training of all staff in euthanasia methods was confirmed prior to euthanasia being performed. Tissue was stored at − 80 °C until protein extraction was performed. Proteins were isolated from the ipsilateral (right) and contralateral (left) cortex and hippocampus using RIPA buffer (100 mM Tris pH 7.5, 150 mM NaCl pH 7.5, 1% NP40, 0.5% sodium deoxicholate, 0.2% SDS, 10 μM aprotinin, 10 μM leupeptin, 1 mM PMSF, 5 mM EDTA, 10 mM Na_3_VO_4_ and 10 mM NaF) containing protease and HALT phosphatase inhibitors (ThermoFisher Scientific, San Jose, CA), 14 days post-injury. Samples were with homogenized and sonicatied followed by centrifugation at 21,000*g* and 4 °C for 20 min. Protein concentration was quantified by BCA assay (Bio-Rad, Hercules, CA). Samples were mixed with 4X loading buffer (Invitrogen, Carlsbad, CA) with 6% β-mercaptoethanol heated for 5 min at 70 °C and loaded in NuPAGE 4–12% Bis–Tris Gel (Invitrogen, Carlsbad, CA). The resulting gels were transferred to a PVDF membrane that was blocked in 0.2% I-Block/PBST for 1 h at room temperature, and incubated overnight with the primary antibody diluted in PBST with 0.2% I-Block (ThermoFisher Scientific, San Jose, CA). After 3 washes in PBST, the membrane was incubated for 1 h with the proper secondary antibody conjugated with diluted horseradish peroxidase. The membranes were washed 4 times in PBST and incubated with ECL substrate (Pierce, San Jose, CA)) then exposed with X-ray film. Primary Antibodies used for the protein detection are: Anti-phospho-Akt (Cell Signaling, Danvers, MA, # 9271) (1:1000), anti-phospho-GSK3β (Cell Signaling #9336) (1:1000), anti-phospho-Erk1/2 (Cell Signaling #4377) (1:1000) anti-Akt (Cell Signaling #9272), anti-GSK3β (Cell Signaling # 9315)(1:2000), anti-Erk (Zymed, San Francisco, CA, #71-1800) (1:5000), anti-β-catenin (Santa Cruz Biotechnology, Santa Cruz, CA #SC-7199) (1:1000), anti-PARP (Cell Signaling #9542) (1:1000), anti-LC3B (NOVUS, St. Louis, MO, #100-2200) (1:4000), and β-actin (Santa Cruz Biotechnology, Santa Cruz, CA #SC-47778) (1:6000).

### Dendritic analysis

Mice anesthetized with isoflurane and perfused with phosphate buffered saline followed by 4% neutral buffered formalin 30 days post injury. Whole brains were placed in 10% neutral buffered formalin overnight at 4 °C. Brains were then cryoprotected in 15% sucrose for an additional 24 h. Cortical samples were incorporated into formalin-fixed tissue blocks (2–3 mm thick in the coronal plane) and were stained by the Rapid Golgi method. Amount and distribution of dendritic branching was evaluated using Sholl analysis and complexity of the dendritic arbor using branch point analysis as previously described [32].

Fixed tissue blocks were initially placed in potassium dichromate and osmium tetroxide for approximately 6 days, then transferred to 0.75% silver nitrate for approximately 40 h. Blocks were then dehydrated through increasing concentration of alcohol solutions and ethyl ether, and infiltrated with increasing concentrations of nitrocellulose solutions (5%, 10%, 20%, 30%; 1–2 days each), placed in plastic molds, and hardened by exposure to chloroform vapors. Tissue sections were to a thickness of 120 microns in the coronal plane using an AO sliding microtome, cleared in alpha-terpineol, rinsed with xylene, and mounted on slides using Permount. Neurons selected for dendritic analysis had to meet strict criteria. Golgi stained neurons randomly selected had to be well impregnated; branches had to be unobscured by other neurons or their dendrites, glia, blood vessels, or undefined precipitate (and staining by-product), and the soma had to be located in the middle third of the thickness of the section. A Zeiss bright field microscope with long-working distance oil-immersion objective lessee and drawing tubes was used to prepare camera lucida drawings.

Dendritic arbors were analyzed using either of two methods: the Sholl Analysis, which defined the amount and distribution of the dendritic arbor, as well as an estimate of the total dendritic length, and the dendritic Branch Point Analysis (BPA) which characterized the complexity of the arbor based on the number of branch points and dendritic bifurcations within the dendritic domain. Additionally, the area of the soma of each neuron was measured using a digitizing tablet linked to the drawing tube of the microscope. All observations were performed consistently by the same blinded observer across samples. Prism software was used to analyze all data statistically.

### Statistical analysis

Mean values are depicted ± standard deviation and were compared with the two tailed t test or ANOVA, as indicated in the legends, with *p *< 0.05 indicating significance. For Sholl and Branch Point Analyses, the Wilcoxon rank-sign test was used to compare dendritic branching profiles of the two groups. When appropriate, an adjusted alpha level was used to account for multiple comparisons.

## Results

### Animal health

No significant weight loss was noted during the experiment and there was no significant difference in weight between groups. Animals remained healthy throughout the duration of the experiment.

### Behavior

**Fig. 2:**
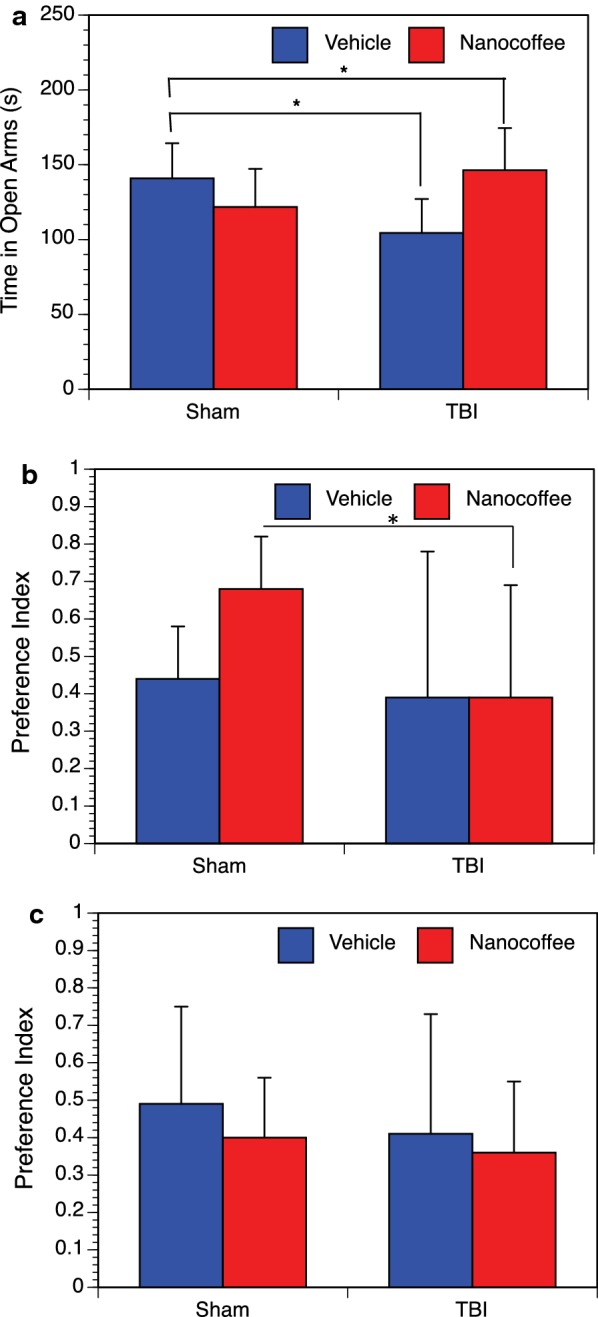
Elevated Plus Maze and Novel Object Recognition. Anxiety-like behavior and recognition memory were evaluated. **a** During Elevated Plus Maze testing a significant effect was seen with reduced time in the open arms of the plus maze after TBI (*p* = 0.016) and this was reversed by treatment with nanocoffee (*p* = 0.013). **b** Novel Object Recognition performed at 14 days post-injury showed that sham mice treated with nanocoffee displayed a greater preference for the novel object than all other groups (*p* = 0.044, when compared to TBI mice receiving nanocoffee). **c** When Novel Object Recognition was performed at 23 days, no significant differences between groups were observed

In the elevated plus maze test, mice were allowed to explore an apparatus with two open and two closed arms. Seven days post-injury, we found that within vehicle treated mice, injury resulted in a significant decrease in the amount of time spent in the open arms, indicating an increase in anxiety-like behaviors (Fig. 2a). When injured mice were treated with nanocoffee, the opposite effect was observed. Mice receiving nanocoffee following TBI spent significantly more time in the open arms than their sham counterparts. This indicates that the treatment may have a positive effect on the anxiety associated with TBI.

Novel object recognition was performed starting at 14 and 23 days post-injury. At 14 days post-injury sham mice treated with nanocoffee showed a greater preference for the novel object than all other groups (Fig. 2b). This suggests that coffee alone may have an enhancing effect on recognition memory, but that this effect is reversed by TBI. At 23 days post-injury, this trend was not observed (Fig. 2c).

### Protein analysis

**Fig. 3:**
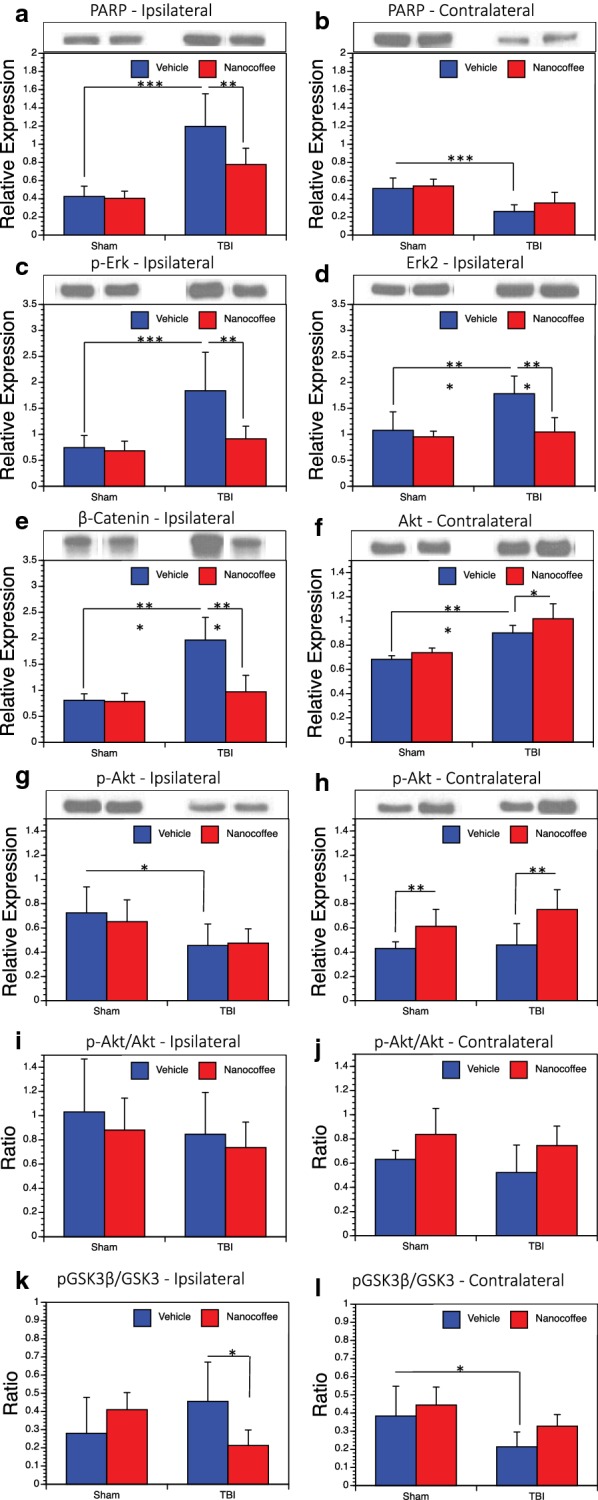
Protein levels. Western blots were used to assess protein levels within the cortex and hippocampus on both the ipsilateral (right) and contralateral (left) sides of the brain relative to the injury. Quantification is performed and expression levels relative to β-actin are shown. **a** PARP was increased in vehicle treated TBI mice in the ipsilateral hemisphere. **b** In the contralateral hemisphere, the opposite effect was observed. p-Erk (**c**), Erk2 (**d**), and β-catenin (**e**) all shown increases in vehicle treated TBI mice on the ipsilateral side. On the contralateral side, Akt (**f**) was increased with injury and further increased by treatment. p-Akt was decreased with injury in the ipsilateral hemisphere (**g**) and increased with treatment in the contralateral hemisphere (**h**). No significicant changes were observed in the p-Akt/Akt ratio on the ipsilateral side (**i**) or the contralateral side (**j**). Finally, pGSK3β/GSK3 ratio was decreased with treatment in injured mice in right hippocampus (**k**) and decreased with injury in left hippocampus (**l**)

We analyzed changes in protein expression in both the ipsilateral and contralateral hippocampus via western blot to characterize the molecular effects of both TBI and nanocoffee treatment 14 days post-injury. We found several significant changes in protein expression within these regions. In ipsilateral hippocampus, we found that PARP expression is significantly increased with injury, but that this increase is significantly reduced by treatment with nanocoffee (Fig. 3a). On the contralateral side, the opposite effect was observed, poly (ADP-ribose) polymerase (PARP) was decreased with injury, but treatment lessened the decrease (Fig. 3b). Phosphorylated extracellular signal-related kinase (p-Erk) (Fig. 3c), extracellular signal-related kinase 2 (Erk2) (Fig. 3d), and β-catenin (Fig. 3e) expression were significantly increased by injury, but not when treated with nanocoffee in the ipsilateral hippocampus. No significant trends were observed in contralateral hippocampus (not shown). No trends were observed in ipsilateral hippocampus for protein kinase B (Akt) expression (not shown), however, contralateral Akt was increased with injury in both treated and vehicle groups (Fig. 3f). P-Akt showed a modest decrease in expression following injury, which was not ameliorated by treatment on the ipsilateral side (Fig. 3g). Contralateral p-Akt was increased by injury and further increased by treatment in both the injury and sham groups, though injury did not appear to have an effect on its own (Fig. 3h). No significant trends were observed in the ratio of p-Akt to Akt, ipsilaterally (Fig. 3i) or contralaterally (Fig. 3j). The pGSK3β/GSK3β (glycogen synthase kinase 3 beta) ratio was significantly reduced by treatment in injured ipsilateral hippocampus (Fig. 3k). In contralateral hippocampus this ratio was significantly decreased with injury (Fig. 3l). In both cases, the effect of injury itself was not significant. No notable trends were observed in microtubule-associated protein 1A/1B-light chain 3 (LC3) levels (not shown).

### Golgi staining and analysis

**Fig. 4:**
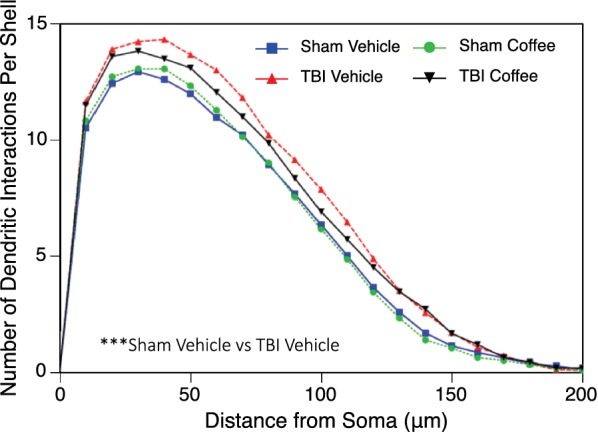
Dendritic arbor analysis. Sholl analysis of Golgi stained pyramidal neurons from layer V of the parietal cortex indicated that the TBI resulted in greater dendritic complexity, especially for the proximal regions, compared to the sham injured groups and the injured group that received nanocoffee (*p *= 0.0003). Sham Vehicle, TBI Vehicle, Sham Nanocoffee groups had 7 mice and 42 total neurons analyzed; TBI Nanocoffee had 6 mice and 37 total neurons analyzed

**Fig. 5:**
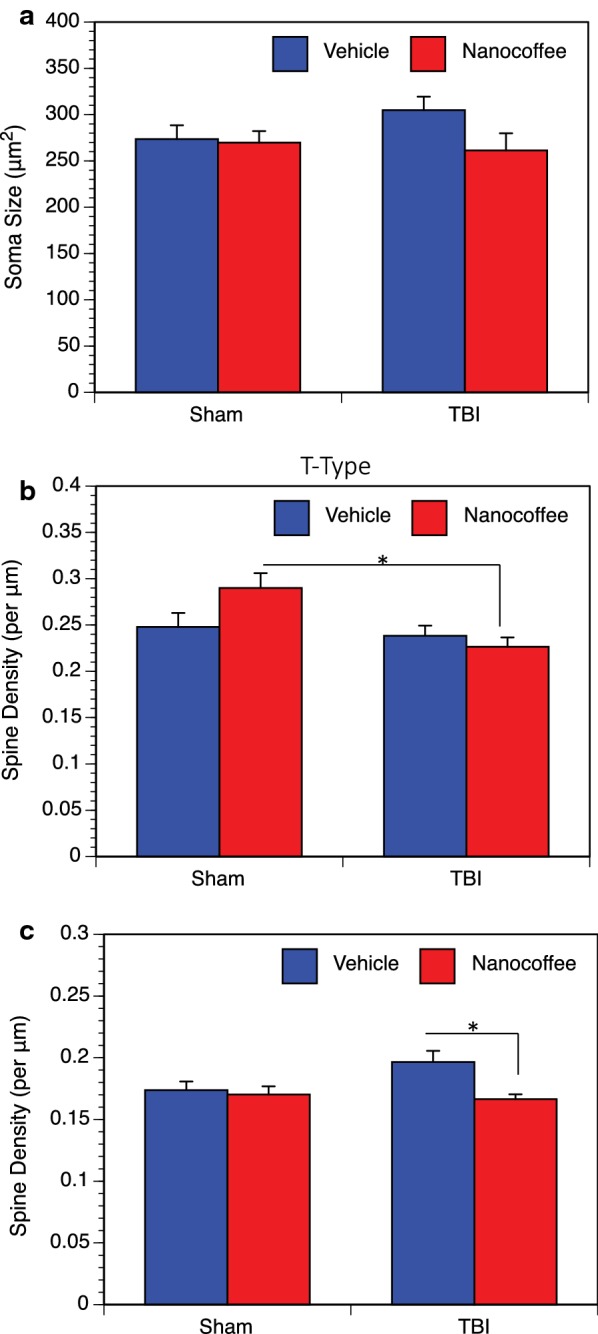
Soma size and spine densities. **a** Soma size was increased by 11% in untreated TBI mice relative to sham mice, though this result was not statistically significant. No changes were observed in mice treated with nanocoffee. **b** T-type spines were elevated in density in the uninjured, nanocoffee treated group (*p* = 0.41). **c** D-type spines displayed a 13% increase after TBI that was reversed back to sham levels by the nanocoffee treatment (*p* = 0.046). For the Sham Vehicle group, 5 mice were analyzed and 34 total neurons. For the Sham Nanocoffee group, 4 mice and 28 total neurons were analyzed. For the TBI Vehicle and Nanocoffee groups, 4 mice and 25 total neurons were analyzed

Sholl analysis was used to assess the distribution of the dendritic arbor within the layer V pyramids of the parietal cortex 30 days post-injury. In our analysis, we found that there was a significant increase in dendritic interactions per shell with injury in vehicle treated mice. We also found a trending decrease in the size of the dendritic domain when TBI mice are treated with nanocoffee particles (Fig. 4). In addition to this, we found an observable, but not statistically significant, 11% increase in soma size in TBI mice treated with the vehicle over all other groups (Fig. 5a). Mice treated with coffee nanoparticles, without TBI, show a 13% increase in dendritic length in cortical layer V pyramids. While there was no significant increase in total spine density (not shown), coffee treated sham mice showed a 17% increase in T-type (“thin”) spines relative to coffee treated TBI mice, which are believed to be associated with learning [33–35] (Fig. 5b). Conversely, untreated TBI mice had a 13% increase in D-type (“dimple”) spines when compared to coffee treated TBI mice (Fig. 5c). These may be reflective of small degenerating spines [32, 36, 37]. Branch point analysis was also performed, but did not reveal any significant differences between groups (not shown).

## Discussion

Our TBI model investigated whether the neuroprotective effects of coffee observed in neurodegenerative disease studies can also be observed in mild traumatic brain injury. This model TBI was followed by treatment by injection of nanocoffee particles. Behaviorally, we saw increases in anxiety-like behavior in the elevated plus maze 14 days post injury when compared to sham controls. This increase in anxiety was not observed when mice were treated with nanocoffee, suggesting that treatment may help to prevent the onset of anxiety associated with brain injury. We also observed that mice had increased recognition memory when treated with coffee, without injury, in the novel object recognition task when compared to uninjured untreated controls. This was not observed when coffee treated mice had previously received an injury. This suggests that, while injury alone did not seem to impact recognition memory, injury may serve to dampen the memory-heightening effects which have been noted previously with coffee treatment [19, 20].

We utilized western blot to analyze the biochemical changes within both ipsilateral and contralateral hippocampus following injury and nanocoffee treatment. Unsurprisingly, PARP was increased with injury in ipsilateral hippocampus. This has been shown previously in brain injured humans [38, 39]. Inhibition of PARP following injury has been shown to reduce microglial activation and neurological deficits in animal models of traumatic brain injury [40, 41]. Moreover, caffeine metabolites have been identified as inhibitors of PARP activity at physiological concentrations [42], suggesting that consumption off coffee, or treatment with coffee extracts such as ours, may effectively inhibit PARP following brain injury. Our data supports this, as PARP was decreased in ipsilateral hippocampus in injured mice following treatment with nanocoffee.

We see a similar pattern of expression for both p-Erk and Erk2. Like PARP, Erk and other members of the mitogen-activated protein kinase (MAPK) signaling pathway have been implicated in the mediation of secondary injury following trauma [43, 44]. Numerous studies have also identified caffeine and other compounds found in coffee as potential inhibitors of Erk expression [45] and phosphorylation and potentially beneficial in the treatment of neurodegenerative disease [46] and cancer [47]. Our nanocoffee treatment supports these previous findings.

β-catenin has been shown previously to be induced following traumatic brain injury and may play a role in vascular repair [48]. We saw an increase in β-catenin following injury and a subsequent decrease with nanocoffee treatment. Little is known about the effect of coffee on β-catenin accumulation or activity, however, our result suggests that coffee, or some compound within coffee, may prove detrimental to the repair processes initiated by β-catenin or the Wnt signaling pathway. However, we do see increases with injury, followed by decreases with treatment, in another Wnt signaling associated kinase, phosphorylated GSK3. This kinase has a multitude of functions, but has been implicated in the progression of injury-associated damage and marked as an emerging target for inhibition to treat TBI [49]. Overall, our results support previous studies and suggest that treatment with a nanocoffee extract may be an effective means of treating TBI by impacting several damage-associated pathways synergistically.

Golgi staining was performed on parietal cortex 30 days post-injury and revealed an interesting increase in the distribution of the dendritic arbor in untreated TBI mice via Sholl analysis. This is a phenomenon that we have seen using this closed head injury model previously [15]. This may be the result of compensatory dendritic hypertrophy, where an increase in connectivity occurs as part of a recovery response. In the 30 days following injury, it is possible that the significantly injured neurons have been eliminated and the remaining neurons have increased their dendritic arbors to compensate for the loss. Also possible is that this is the result of selective loss. Neurons that were already less distributed were lost following injury while more complex neurons survived, resulting in an overall increase in average distribution via the Sholl analysis. It is interesting that we did not see this increase in dendritic arbor distribution when injured mice were treated with nanocoffee particles. This could be due to decreased compensatory hypertrophy due to neurons having sustained less damage overall and may suggest a neuroprotective effect of the nanocoffee treatment. This is further supported by the increased soma size that was observed in the untreated TBI mice. Hypertrophied cell bodies have been shown to be indicative of neuronal damage [50]. Given that we did not see an increase in soma size in nanocoffee treated mice, this would support our hypothesis that there is more long-term damage being sustained by the neurons in TBI mice that are untreated. The same pattern was observed when we analyzed the small degenerating D-type (dimple) dendritic spines in untreated and treated TBI mice; again, indicative of more damage being sustained in untreated mice.

## Conclusion

We have taken a multifaceted approach to the investigation of a coffee extract in the treatment of mild traumatic brain injury. We have identified coffee treatment as having positive effects behaviorally, biochemically, and morphologically. Our data suggest that injection of coffee extracts in the period following a traumatic brain injury may be effective at ameliorating cognitive deficits and improving overall neuronal health and recovery.

## Data Availability

The datasets generated during and/or analyzed during the current study are available from the corresponding author on reasonable request.

## Abbreviations

TBI: traumatic brain injury
mTBI: mild traumatic brain injury
AD: Alzheimer’s disease
Aβ: amyloid beta
TDP-43: TAR DNA binding protein 43
GABA: gamma-aminobutyric acid
NFκB: nuclear factor kappa-light-chain-enhancer of activated B cells
GSK-3α: glycogen synthase kinase 3 alpha
(p-)Akt: (phosphorylated) protein kinase b
(p-)GSK3β: (phosphorylated) glycogen synthase kinase 3 beta
(p-)Erk1/2: (phosphorylated) extracellular signal related kinase ½
PARP: poly-ADP ribose polymerase
LC3: microtubule-associated protein 1A/1B-light chain 3
